# Impact of Health Research Capacity Strengthening in Low- and Middle-Income Countries: The Case of WHO/TDR Programmes

**DOI:** 10.1371/journal.pntd.0001351

**Published:** 2011-10-11

**Authors:** Happiness Minja, Christian Nsanzabana, Christine Maure, Axel Hoffmann, Susan Rumisha, Olumide Ogundahunsi, Fabio Zicker, Marcel Tanner, Pascal Launois

**Affiliations:** 1 Ifakara Health Institute, Ifakara, Tanzania; 2 Swiss Tropical and Public Health Institute, Basel, Switzerland; 3 University of Basel, Basel, Switzerland; 4 UNICEF/UNDP/World Bank/WHO Special Programme for Research and Training in Tropical Diseases, Geneva, Switzerland; University of Ottawa, Canada

## Abstract

**Background:**

Measuring the impact of capacity strengthening support is a priority for the international development community. Several frameworks exist for monitoring and evaluating funding results and modalities. Based on its long history of support, we report on the impact of individual and institutional capacity strengthening programmes conducted by the UNICEF/UNDP/World Bank/WHO Special Programme for Research and Training in Tropical Diseases (TDR) and on the factors that influenced the outcome of its Research Capacity Strengthening (RCS) activities.

**Methodology and Principal Findings:**

A mix of qualitative and quantitative methods (questionnaires and in-depth interviews) was applied to a selected group of 128 individual and 20 institutional capacity development grant recipients that completed their training/projects between 2000 and 2008. A semi-structured interview was also conducted on site with scientists from four institutions. Most of the grantees, both individual and institutional, reported beneficial results from the grant. However, glaring inequities stemming from gender imbalances and a language bias towards English were identified. The study showed that skills improvement through training contributed to better formulation of research proposals, but not necessarily to improved project implementation or communication of results. Appreciation of the institutional grants' impact varied among recipient countries. The least developed countries saw the programmes as essential for supporting basic infrastructure and activities. Advanced developing countries perceived the research grants as complementary to available resources, and particularly suitable for junior researchers who were not yet able to compete for major international grants.

**Conclusion:**

The study highlights the need for a more equitable process to improve the effectiveness of health research capacity strengthening activities. Support should be tailored to the existing research capacity in disease endemic countries and should focus on strengthening national health research systems, particularly in the least developing countries. The engagement of stakeholders at country level would facilitate the design of more specific and comprehensive strategies based on local needs.

## Author Summary

The UNICEF/UNDP/World Bank /WHO Special Programme for Research and Training in Tropical Diseases (TDR) has over the 2000–2008 period supported the development of individual and institutional grants. Although the TDR research capacity development programmes has had a substantial impact on the development of tropical disease research and research capacity in disease endemic countries, a review of the lessons learnt and benefits of this approach has never been completed. A study was conducted to analyse TDR's inputs in research capacity in endemic countries and to assist TDR in the improvement of its future activities. An analysis (by variables of gender, age, language, country of origin, country of studies, type of grant, scientific interest etc) of the grantees that have benefited from TDR support in terms of their career development and research capacity, including any important financial implications was conducted. The study identify opportunities that are a broader relevance to objectives to international development agencies such as addressing inequities such as the gender imbalance language bias towards English and building a supportive research environment in DECs in which researchers can develop their scientific career and pursue their research.

## Introduction

Health research capacity is unanimously recognized as contributing to the overall development of low-and middle-income countries and is a critical precondition for achieving the Millennium Development Goals [1], [2].

Research capacity strengthening (RCS) is defined as “the process by which individuals, organizations and societies develop ability (individually and collectively) to perform functions effectively, efficiently and in a sustainable manner to define objectives and priorities, build sustainable institutions and bring solutions to key national problems” [3].

Health research capacity strengthening programmes have been identified as a driver for the support of international development agencies [4]. Although these programmes created a large number of well-trained health researchers and institutions, and despite the remarkable progress made by some low- and middle-income countries in engaging in their own capacity building, health research capacity strengthening remains a challenge, particularly in sub-Saharan Africa [5]. This can be attributed to the limited ability of development agencies to identify, target and influence necessary factors that lead to an effective, efficient and relevant RCS programme in health, despite the availability of several frameworks for monitoring and evaluating RCS results and modalities of funding [4], [6]–[9]. Indeed, evaluating health RCS initiatives is quite complex, since achieving the objectives could take several years (often more than 10 years). However, evaluation is necessary to provide information to justify the (dis) continuation of programmes and to highlight the areas that need improvement [4], [10].

Among the organizations with extensive RCS experience is the UNICEF/UNDP/World Bank/WHO Special Programme for Research and Training in Tropical Diseases (TDR). Created in 1975 to support the research and capacity building in the fight against infectious diseases of the poor, TDR goal is to improve health and remove diseases as barriers to social and economic development. For more than 30 years, TDR has built health research capacities in developing countries by supporting individuals' education and training through fellowships or scholarships; implementing learning by doing programmes for specific skills; employing mentorship programmes to complement academic programmes; establishing national and international training and research centres of excellence; and developing networks and collaborative research projects.

Regular reviews of its research capacity strengthening programmes have led TDR to reorient its strategy as required: shifting focus from institutional strengthening in the 80 s to human resources strengthening in the 90 s [11] and identifying the need to move beyond the idea of RCS as being primarily related to individual researchers to a more demand driven model of national health research systems [12]. TDR models of capacity building and particularly the merit of short-term trainings in social sciences have also been evaluated [13] but still, there has been no systematic and comprehensive data of the lessons learnt and benefits of the different TDR RCS approaches i.e. individual and institutional. Thus, we conducted an evaluation of TDR's contribution to career strengthening of a selected group of individuals and institutional capacity development grantees with a record of project completion between 2000–2008. The main objective was to identify factors that positively influenced and improved the research capacity and career development of grant recipients for identifying opportunities that are of broader relevance to the objectives and goals of international development and aid agencies.

## Methods

Between 2000 and 2008, TDR supported 128 individual grants -including 88 research training grants (RTG), 40 re-entry grants (REG) and 20 institution strengthening grants (ISGs) that were completed during the same period. RTGs are awarded to individuals in developing countries to pursue studies leading to acquisition of a postgraduate degree (MSc or PhD) at institutions in their home countries, or in other developing or developed countries. REGs are intended to facilitate the career development of young scientists returning to their home institutions within 24 months following completion of a graduate degree (MSc or PhD) or a post doctoral fellowship. ISGs are designed to provide up to three years support to an institution or research group to improve infrastructure and the research environment. Activities may include improving training opportunities, advancing scientific expertise in biomedical and social sciences, establishing/improving information and communication systems, and fostering opportunities for scientific collaboration.

Information on all individuals and institutions that received TDR grants between 2000 and 2008 was extracted from TDR internal database and tabulated for range and scope of research topics.

A mix of quantitative and qualitative methods was applied during data collection and analysis to capitalize on the advantages and minimize the limitations of each approach. The assessment consisted of three standardized questionnaires sent by e-mail to those individuals that completed their project within the 2000–2008 period. The first questionnaire was sent to recipients of research training grants to assess their career progression, the skills acquired during their training, and the impact of the training on the research capacity of their home institution. A second questionnaire was sent to individuals who received a re-entry grant to assess the performance of their research group and the impact of their research on the development of their institutions. A third questionnaire was sent to the principal investigator of institutions that received TDR grants to assess the impact of the grants on institutional performance.

Individual questionnaires were designed to obtain information on the current position of each grantee, and assess the research competencies of each individual, both before and after the TDR grant period, for comparison purposes. Additionally, the questionnaires were also designed to self evaluate the following five main indicators prior to, during, and after the grant period: scientific publications; ability to attract additional resources; participation in national and international collaborative activities; human resource development, including staff development and training and provision of research equipment by the home institution.

A total of 10 RTG recipients were selected for in-depth interviews. Interviewees were selected so as to achieve gender balance and representation from a variety of research interests and countries of origin. The interviews aimed to collect information about the grantees' perspectives on the factors influencing their careers after the training grants. Opinions on how to improve TDR research capacity strengthening programmes to meet the needs of disease endemic countries and their populations were also collected.

Questionnaires for institutions included a self-assessment of the following institutional performance indicators: work space; library; internet and e-mail access; laboratory facilities; purchasing and inventory systems; maintenance and repair facilities and human resources.

Taking into account a balance of research topics and regional representation, four institutions were selected for site visits. A semi-structured interview was conducted with the leaders and scientists of each institution. Interviewees were asked about their views on the following issues: the strengths and weaknesses of TDR funded institutions; the contribution of these institutions to formulation of national public health policies; the extent to which these institutions satisfied stakeholders' ongoing requirements for (access to) quality goods and services.

Data from both individual and institutional questionnaires was analysed using STATA (version 10), based on a prepared data dictionary. The in-depth interviews were tape-recorded, transcribed and imported into a pre-coded template prepared in Microsoft Word for export to MaxQDA. The main findings are illustrated with selected short narratives.

## Results

### Analysis of TDR grantees

Analysis of TDR grants by age and sex of recipient; regional and language distribution; and research area is given in Table 1.

**Table 1:** Analysis of the TDR grants during the 2000–2008 period.

Type of grants	RTGs*	REGs*	ISGs*
Number of grants	116	83	41
Number of grants completed (%)	88 (75.9)	40 (48.2)	20 (48.8)
Age (mean ± sd in years)	33±4.5	36±4.2	NA
% of women	28	41	NA
Regional distribution (WHO regions in % of grants)			
AFRO	65.5	37.5	49.0
AMRO	8.5	38.5	24.5
EMRO	6.0	6.0	5.0
SEARO	23.0	15.0	9.5
WPRO	7.0	4.0	9.5
Language distribution (% of grants)			
English	65.5	38.5	46.5
French	15.5	20.5	24.5
Spanish	6.0	15.5	12.0
Portuguese	6.0	23.0	12.0
Chinese	3.5	2.5	-
Arabic	3.5	-	5.0
Research area (% of grants)			
Epidemiology	42.5	15.5	32.0
Basic sciences	21.5	49.5	24.5
Social sciences	15.5	3.5	12.0
Entomology	12.0	18.0	5.0
Product development	7.5	9.0	12.0
Clinical investigation	5.0	3.5	12.0

NA not applicable.

*RTG are awarded to individuals to pursue studies leading to the acquisition of a postgraduate degree (MSc, PhD).

*REG are intended to facilitate the career development of young scientists returning to their home institutions within 24 months following completion of a graduate degree (MSc, PhD).

*ISG are intended to provide long-term (three years) support to institution.

The mean age of RTG recipients is 33±4.5 years, with women representing 28% of all grantees. Most RTG recipients are from Africa (65.5%), and mostly from Anglophone countries (65.5%). The majority funded projects were on epidemiology and disease control (42.5%), while social sciences represented 15.5% of the projects. Of the 88 questionnaires sent to recipients of TDR RTGs, 59 (67%) were returned. Of those that responded, 33 received a PhD, 23 an MSc and three participated in a short training course.

The overwhelming majority (82%) of grantees moved abroad for their training with a mean period of 21 months, a minimum of two months and a maximum of 72 months. MSc candidates preferred to study at the University of Witwatersrand in South Africa (26%); the London School of Hygiene and Tropical Medicine (LSHTM) in the UK (12%); or the Liverpool School of Tropical Medicine (LSTM) (12%) in the UK. PhD trainees moved to LSHTM (8.9%), the LSTM (8.9%), and the Swiss Tropical and Public Health Institute (formerly Swiss Tropical Institute) Institute (6.6%) in Switzerland. A few (6.6%) of PhD grant recipients received their doctoral training at institutions in their country of origin.

One grantee's view on moving abroad for training indicates a gender bias as expressed in the following quote: “*I do not want to speak of my case as special but it is still a man's world and this is reality. When a man gets an opportunity such as a training grant and has to go abroad he will not think twice he will just get up and go. But a woman’s reality is different meaning that social responsibilities make her not as flexible as her fellow partner. If TDR capacity strengthening programmes do not have this understanding in their philosophy, then most women will have troubles to join such programs*” (Female grantee).

The mean age of REG recipients was 36±4.2 years with women representing 41% of all grantees. REGs were predominantly awarded to scientists from Africa (37.5%) and South America (38.5%) and English was the most common language used by the grantees (38.5%). Of the 40 questionnaires sent to recipients of TDR REGs, 25 (62.5%) were returned. Of those that responded, 60% (15/25) were based in universities, 32% (8/25) in research institutes, and 8% (2/25) in governmental agencies.

Most of the ISGs were awarded to Anglophone countries (46.5%) and institutions from sub-Saharan Africa (49%). A Francophone scientist reported that, “*language, in my case English language, was an important barrier preventing me and my fellow scientists from applying to competitive international grants, including those from TDR*” (Male grantee).

The majority of the projects funded focused on epidemiology and disease control (35%) and basic research (25%). Of the 20 questionnaires sent to ISG recipients, eight were returned; five from research institutes and three from universities.

### RTG recipients' research skills developed during the training

Three categories of research competencies were identified for self-assessment:

1. Developing a research project based on the ability to identify a research problem; conduct a scientific review; analytically review a scientific article; and write a research proposal.
2. Conducting a research project based on the ability to methodologically conduct situational analysis; implement and manage a research project; manage and analyse data; and interpret scientific data.
3. Communicating scientific findings based on the ability to communicate information to scientific communities, stakeholders, and the general public.

The level of each research competency was self -assessed and graded on a scale of 1 to 7 (1 being poor or no competency and 7 for very good or high competency). Most RTG recipients (74%) rated their research competencies as very good (grade of 6 or 7), and 24% considered themselves to have medium level skills (grade of 3 to 5). 58% of the grantees rated themselves very good in conducting situational analysis and 90% of grantees cited the ability to write in English as the most important skill acquired during training.

**Figure 1:**
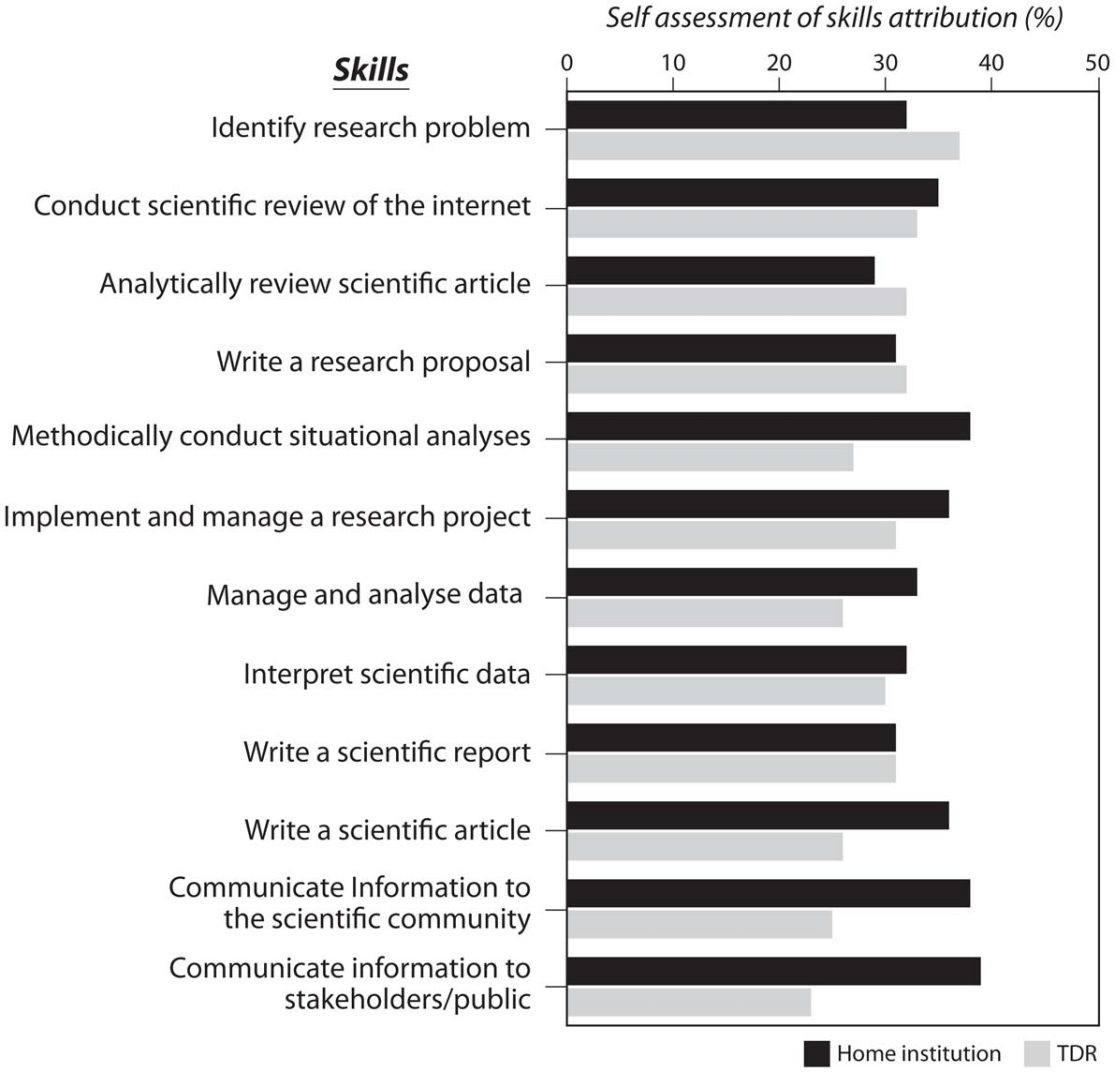
Individual grantee skill competency attributed to TDR. An estimation of the percentage of attribution to the home institution and to TDR of the different competencies was assessed by individual. Results are presented as the percentage of attribution of each competency to TDR and to the home institution. TDR attribution was considered to be moderate or low when it was lower than the home institution attribution.

Grantees also assessed the attribution of the acquired skills to TDR funding or to the home institution. Results in Figure 1 show that TDR training is perceived to have contributed to better formulation of a research proposal, but not necessarily to an improved ability to conduct a research project and communicate research results to a broad audience. The following statement reinforces this finding: “*When TDR provides research training support to grantees, they assume those candidates have the necessary research skills. TDR gives you money to go collect data analyse and write report, but for me that is not enough. The skills that I would consider most important are how to manage the grant itself, how to implement the research activity, analyse data and write the report once the grant has been successfully managed*” (Male grantee).

The analysis also shows that the percentage of grantees that received a competitive grant after completing a TDR-funded training increased from 20% to 34%. The average number of competitive grants obtained by each individual increased from 1.61 to 2. Of those who had not received a competitive grant prior to TDR-funded training, 42% obtained research grants post training. Similarly, the number of publications increased from 3 fold, with an average of 30 citations in the post-TDR grant period, based on a Medline search of publications and citations of all grantees. The following grantee narrative highlights issues regarding scientific publications: “*Before receiving research training support, it was difficult to publish as a candidate does not have what it takes to publish in a peer reviewed journal…publishing is a different world and requires advanced writing skills, confidence to write, and knowledge about the publishing process, which for many scientists in developing countries is missing*” (Male grantee).

### Impact of REGs on research groups or institutions

**Figure 2:**
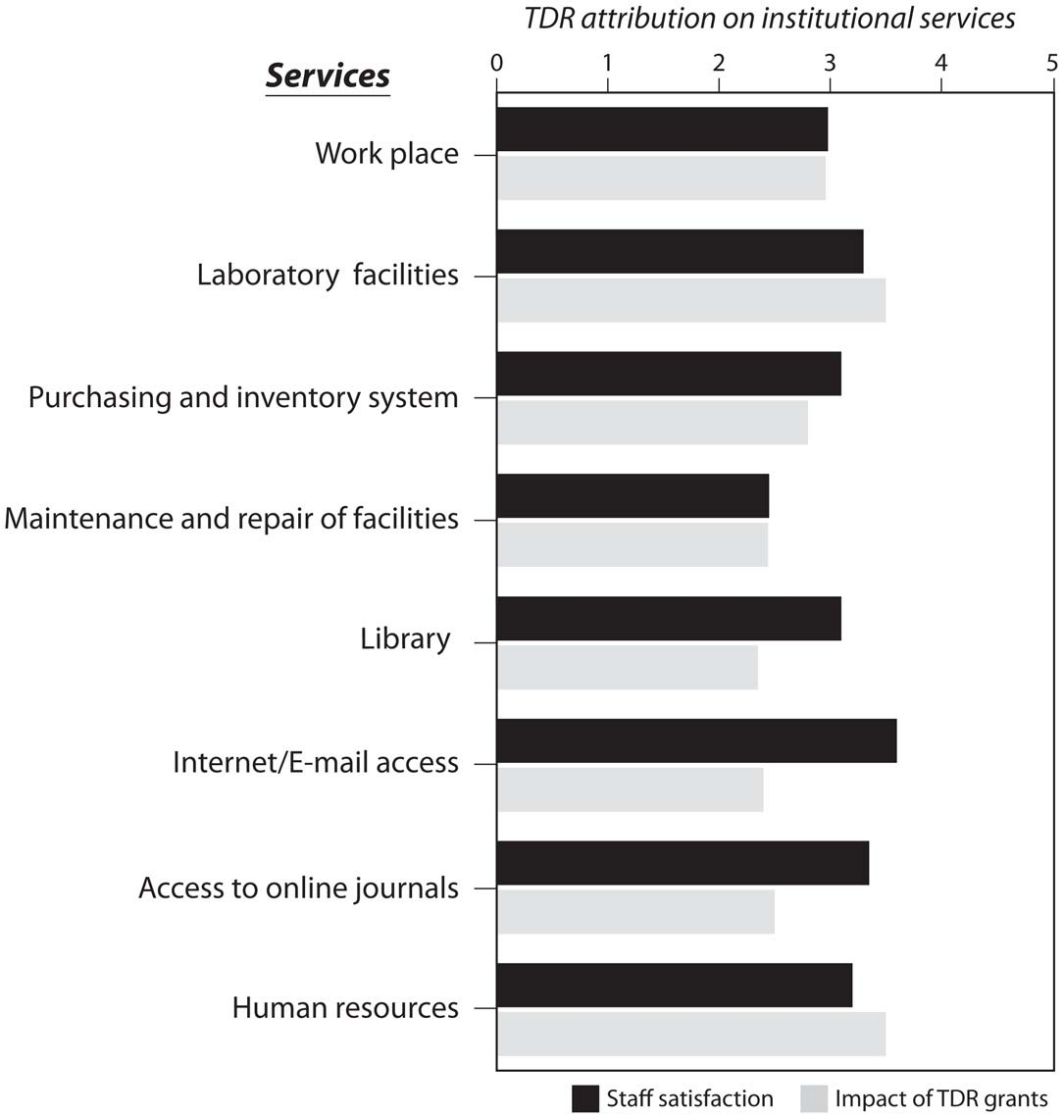
REG recipient attribution of institution service improvements to TDR. TDR attribution to the improvement of the institution's services was assessed. Ranking was defined as follows: 1 = none, 3 = moderate and 5 = high.

Analysing the responses from REG recipients allowed us to assess the level of satisfaction with the following services: work place; library; internet access; access to online journals; laboratory facilities; purchasing and inventory system; maintenance and repair of facilities and human resources. The responses also allowed for attribution of improved work place amenities and services to TDR training. Figure 2 shows that TDR grants were seen to have the highest impact on the work place, laboratory facilities, human resources and maintenance and repair of the equipment. TDR was however considered to have only a moderate impact on library services, access to internet, and access to online scientific journals.

As with RTGs, the analysis of REGs showed that the number of competitive grants increased: 85% of all research groups received grants after completing TDR REG grant compared to 67% previously. Similarly, an average of three grants per group was received post TDR support, compared to two grants per group before. Regarding the benefits to the home institution, 79% of grantees reported that TDR grants facilitated institutional acquisition of durable equipment, especially laboratory equipment. An average of 11 students (six undergraduate, three MSc and two PhD students), 45% of which were women, were trained through TDR grants and 70% of the research groups reported that they trained or employed at least one technician during TDR training. 78% of the respondents mentioned that they were able to retain at least one technician after TDR training.

### ISG impact on research institutions

TDR ISGs were reported to have a positive impact on all of the services analysed, with the greatest impact being on laboratory facilities and human resources. All institutions surveyed mentioned that they were able to acquire durable laboratory equipment. The following quote from an ISG beneficiary illustrates on this finding, “*TDR contribution to scientific capacity in tropical disease endemic countries is undeniably visible…TDR financed the purchase of the first PCR machine and continues to make huge investments in our lab, enabling subsequent scientific work to be carried out. Without TDR distinctive support, that work would not have taken place and the malaria treatment policy would have been changed based on politics rather than scientific evidence*” (Male grantee).

An average of 53 scientists were trained during the three year support of the ISG. Of those 53, 37% (20 positions: nine undergraduate, six MSc and five PhD students) were directly trained with TDR support. Each institution trained an average of nine technicians and retained at least one after TDR training completion.

The four institutions selected for site visits were the Centre d'Etudes sur les Ressources Végétales (CERVE) in Brazzaville, Congo; the Faculty of Medicine and Health Sciences, University of Sana'a, Yemen; the Centre for Research in Medical Entomology (CRME) in Madurai, India; and the Department of Immunology and Biochemistry, Institute of Biological Sciences, Federal University of Minas Gerais, Belo Horizonte, Brazil. The site visits confirmed that ISG had a substantial impact on human resources development and infrastructure improvements at these institutions. However, appreciation of the ISG's impact varied among countries. Institutions in the least developed countries (Congo and Yemen), saw the ISG as essential to maintaining basic infrastructure and activities, since local authorities do not invest much financial resources in research. Advanced developing countries (India and Brazil), perceived ISGs as complementary to resources received from local authorities, and of particular value to young researchers who were not yet in a position to successfully compete for major international grants.

## Discussion

Some conclusions resulting from the present analysis of individual and institutional TDR capacity building programmes between 2000 and 2008 are relevant for improving and further developing the RCS activities of international development and aid agencies.

First, RCS funding agencies should develop specific strategy to address some health research inequities such as gender imbalance and bias towards Anglophone countries' support. Indeed, in the present study, a pronounced disequilibrium in gender balance was made evident by the fact that only 28% of RTGs and 41% of REGs were allocated to women. In addition, the mean age of the grantees is more than 30 years, indicating that TDR training support coincides with women's prime years for tending to children and related family responsibilities, especially in low-income countries. The data confirms that family responsibilities, particularly child bearing and rearing together with structural and cultural barriers, impinge on women's access to good scientific training. This finding is consistent with other research that asserts that many women with doctorate degree do not work as scientists due to societal biases and structural factors [14], [15]. Thus, to address the under-representation of women in RCS programmes and to promote equity, RCS organizations should develop strategies that are sensitive to the specific situations and needs of women and consequently address overrepresentation of men in the distribution of resources and improve overall research capacity in disease endemic countries.

Most of the TDR grantees came from Africa (65.5%) and most of the grant recipients were Anglophones: 57% of TDR grantees came from English speaking countries, while 20% from French, 10.5% from Portuguese, 8.5% from Spanish, 2% from Chinese, and 1.5% from Arabic speaking countries. Indeed, English is the dominant language in health research and overshadows other languages as a means of communication thereby inadvertently limiting other linguistic communities' access to essential technical information. Poor English language skills hinder wide dissemination of research results by health researchers from disadvantaged populations. As a result, the work of many health researchers in disease endemic countries does not have tangible impact on national, regional or international health research. To overcome the language barriers, health RCS organizations should develop specific strategies, including making appropriate provisions in their grantee selection criteria, to increase the chances of non-Anglophone countries benefitting from their programmes and promoting collaboration between scientists and institutions in more advanced Anglophone countries and their counterparts in less advanced countries. An example is the case of two intergovernmental health organizations, the Francophone Organisation de Coordination et de Coopération pour la Lutte contre les Grandes Endémies (OCCGE) and the Anglophone West African Health Community (AWAC) that merged in 1998, creating the West African Health Organisation (WAHO), an organization committed to transcending linguistic borders to serve all fifteen ECOWAS (Economic Community of West African States). (http://www.wahooas.org/).

Secondly, RCS funding agencies should build and maintain a supportive research environment in DECs in which researchers can develop their scientific career and pursue their research. This strategy includes strong grant management unit in institutions, good communication facilities, career structures and demand from policy markers.

For many institutions in low- and middle-income countries, maintaining a high quality research environment remains a challenge due to limited resources. The present study shows that TDR grants had a great impact on strengthening institutional infrastructure, particularly through acquisition of laboratory equipment (Figure 2) but access to internet/e-mail and online journals remains limited in most institutions. Investment in the necessary infrastructure for high speed/high quality internet access is beyond of TDR RCS's support capacity. Consequently, easy sharing of research information among researchers, media, policy makers, and other public and private stakeholders remains difficult and, at times, costly. Thus, support for capacity building should extend beyond the individual to the institution, through support for equipment acquisition and refurbishment of essential infrastructure.

One objective of the present survey on TDR capacity building was to identify factors that positively influenced research capacity and career development. Developing skills for advocacy, resource generation and allocation, negotiation and consensus building, and financial management were clearly identified as important factors and should be targeted by RCS organizations. Project management skills that are often omitted from academic curricula, have also been identified as critical to develop health research leadership in developing countries. To address this gap, TDR developed a training course on “Effective Project Planning and Evaluation” (EPPE) to help scientists plan, implement, monitor, report and evaluate the progress of research projects in a systematic way. To ensure quality and access to a wider audience, TDR selected and supported a number of institutions to administer the EPPE course locally. These institutions are encouraged to develop national and regional support networks for individual researchers and institutions that serve as mechanisms for sharing ideas and resources. The Centro Internacional de Entrenamiento e Investigaciones Médicas (CIDEIM) in Cali, Colombia is a good example of how a centre of excellence formed a regional network that supports the TDR developed planning, monitoring and evaluation activities in universities and research institutions throughout Latin America and the Caribbean [16]. Communication to the scientific community through peer reviewed scientific journals is equally important to the career development of a researcher. A scientist's eligibility for grants and funding, and general career advancement is closely related to the number of publications he or she produces. As such, many RCS programmes support activities to enhance writing skills and encourage publications. However, it is clear that representation of researchers from low- and middle-income countries in scientific publications remains low. This may be a reflection of poor representation of these countries on the boards of international journals in tropical medicine [17]. Thus, research-funding agencies should consider providing resources to promote the expertise of authors, reviewers and editors from low- and middle-income countries to promote health research publications from underrepresented populations.

Publishing in peer-reviewed journals is not an end in itself, but rather a means of communicating research-generated knowledge which can be translated into health policies, operational guidelines or health products. Translating research results into policy recommendations, concrete interventions or new tools was identified as a major weakness of RCS organizations [18], [19]. The result obtained in the present assessment of TDR programmes confirms this issue. One reason could be that most of TDR RCS grants are for basic medical sciences and epidemiology. These subjects are upstream in the research and development pipeline and the immediate translation of the research into a product is often not possible. The knowledge generated, however, contributes to a better understanding of epidemiology, systems or biology of vectors. Although some RCS organizations recognize the need to bridge the gap between research and policy, more can be done to promote research uptake i.e. synthesizing research results to show new knowledge production and promoting the use of evidence in decision-making. Decision makers at various levels should also be trained on for evidence-based decision-making [20].

In conclusion, health RCS programmes should maintain and expand their investment in training activities to 1) address inequities in health research by taking into account the social and cultural situation of the grantee, 2) introduce criteria that encourage and support the development of research careers within DECs and establish networks and 3) develop country-specific programmes in collaboration with national authorities to address the specific needs of each country, and identify how best to strengthen national health research systems.
